# Quantizing reconstruction losses for improving weather data synthesis

**DOI:** 10.1038/s41598-024-52773-2

**Published:** 2024-02-09

**Authors:** Daniela Szwarcman, Jorge Guevara, Maysa M. G. Macedo, Bianca Zadrozny, Campbell Watson, Laura Rosa, Dario A. B. Oliveira

**Affiliations:** 1 IBM Research, Rio de Janeiro, Brazil; 2 IBM Research, São Paulo, Brazil; 3 IBM Research, Yorktown Heights, NY USA; 4 Laboratory of Geo-information Science and Remote Sensing, Wageningen University and Research, Wageningen, The Netherlands; 5 School of Applied Mathematics, Getulio Vargas Foundation, Rio de Janeiro, Brazil

**Keywords:** Climate sciences, Climate change, Computational science, Computer science

## Abstract

The stochastic synthesis of extreme, rare climate scenarios is vital for risk and resilience models aware of climate change, directly impacting society in different sectors. However, creating high-quality variations of under-represented samples remains a challenge for several generative models. This paper investigates quantizing reconstruction losses for helping variational autoencoders (VAE) better synthesize extreme weather fields from conventional historical training sets. Building on the classical VAE formulation using reconstruction and latent space regularization losses, we propose various histogram-based penalties to the reconstruction loss that explicitly reinforces the model to synthesize under-represented values better. We evaluate our work using precipitation weather fields, where models usually strive to synthesize well extreme precipitation samples. We demonstrate that bringing histogram awareness to the reconstruction loss improves standard VAE performance substantially, especially for extreme weather events.

## Introduction

The frequency, duration, and intensity of extreme weather events have increased as the climate system warms. For example, climate change leads to more evaporation, directly exacerbating droughts and increasing the frequency of heavy rainfall and snowfall events^1^. These extreme weather events often result in hazardous conditions or impacts, either by crossing a critical threshold in a social, ecological, or physical system or co-occurring with other events^2^. In this context, impact models can be valuable tools for risk assessment in different climate-sensitive sectors and for evaluating adaptive measures. However, these models usually require long time series of high-resolution weather data^3^.

Stochastic weather generators are techniques that can create artificial weather series representing plausible climate scenarios^3–5^ and have been widely used to provide data to impact models^6^. Traditional weather generators often use latent weather states and resampling methods. Despite their success in generating realistic weather data, methods relying on resampling usually require careful tuning of spatiotemporal constraints and struggle to scale for large gridded areas or generate diverse samples. Furthermore, traditional weather generators usually struggle to generate extreme events.

Recently, with the great success of deep generative models for many applications^7–9^, researchers have also began investigating these methods in the context of weather field synthesis^10–13^, but most of these works explored only generative adversarial networks (GANs)^14^. PrecipGAN^11^ simulates the spatiotemporal change of precipitation systems, ExGAN^10^ proposes a GAN-based approach to generate realistic extreme precipitation scenarios, and the GAN from Klemmer et al.^13^ generates spatiotemporal weather patterns conditioned on detected extreme events. However, while the GAN adversarial training scheme usually derives efficient losses that lead to generators producing very realistic samples, such models are difficult to train and often present the mode collapse issue, which leads to low diversity synthesis^15,16^.

Variational autoencoders (VAE)^17^ are encoder-decoder generative models that map the training data into a latent distribution and enable stochastic synthesis by merely sampling latent codes from a known (simple) distribution. In the context of extreme precipitation field synthesis, Oliveira et al.^12^ showed that it is possible to control the extremeness of new samples by choosing areas from the known latent space distribution using VAEs.

The loss function for a standard VAE combines a reconstruction term and a regularization term^17,18^. The first is related to the reconstruction error, such as the mean squared error, and the second regularizes the latent space to a known distribution. Several works have investigated different approaches for the regularization term^18–20^, while improvements for the reconstruction term remains mostly underexplored. In regression problems, the mean squared error usually leads to models that show good performance for frequent values and poor results for less common values^21^, especially if the imbalance is severe. However, generating extreme (rare) events is crucial in the context of weather generators, as they are precisely the events of interest for climate change.

In this paper, we propose alternatives to the standard VAE reconstruction loss that uses distributional information of pixel values to improve the model’s reconstruction quality for rare values. More specifically, we propose to penalize the reconstruction loss using histograms of the training data batch and compare the results with strategies used for imbalanced regression datasets. We experiment with our approach using precipitation data, usually highly imbalanced, where rare precipitation values are often associated with extreme precipitation events. We report that our proposed quantized losses substantially improve the reconstruction quality, especially for the highest precipitation values. Concretely, our contributions are listed as below:

- Propose quantized reconstruction losses for improving weather field synthesis reconstruction quality for extreme events;
- Evaluate the proposed approach synthesis quality comparing with baseline benchmarks for handling very imbalanced datasets;
- Report results for reconstruction and stochastic synthesis using real use case data.

## Related work

### **Variational autoencoders**

VAEs^17,22^ are a popular framework for deep generative models and formulate learning representations of high-dimensional distributions as a variational inference problem^23^. The VAE objective balances the quality of generated samples with encouraging the latent space to follow a fixed prior distribution, using a regularization term. Efforts to improve the VAE framework have mainly focused on the regularization part of the objective, like InfoVAE^18^ and Wasserstein autoencoders^19,20^.

More recently, vector quantized variational autoencoders (VQ-VAE)^7^ introduced the quantization of latent space delivering synthesis with improved reconstruction quality, even overcoming the results from many popular GANs^24^. Since the optimized quantized codes are discrete, following a categorical distribution, one cannot use them directly to generate new samples or adjust the synthesis towards extreme scenarios. Van Den Oord et al.^7^ train a PixelCNN using the codes as priors to generate novel examples, which greatly increases the complexity of adjusting stochastic synthesis targets when compared to merely sampling from known areas in the latent distribution as in the standard VAE. Still, it is worth clarifying that our primary contribution in this paper pertains to the utilization of quantized reconstruction losses, rather than promoting a specific VAE architecture.

### Regression in imbalanced training sets

Different solutions have been proposed to improve regression or classification performance for less frequent values in imbalanced datasets. A traditional approach is to define a relevance function that maps the continuous values in the target domain into a scale of relevance, where the infrequent values are the most relevant^25,26^. Another popular strategy increases the model’s exposure to less frequent values by combining both over-sampling and under-sampling strategies^27,28^, even if such strategies can introduce noise or disregard valuable information, respectively. Recently, distributional losses (commonly used for classification and reinforcement learning) were introduced for regression problems^21,29^. Imani et al.^29^ proposed the Histogram Loss (HL) that derives a target distribution that corresponds to a normalized histogram and optimizes the Kullback-Leibler (KL) divergence of prediction and target. Besides improving accuracy, the HL loss function reduces over-fitting and improves generalization. However, the method implements the loss using an extra layer with nodes corresponding to each histogram bin, which limits the loss applicability in any model. Yang et al.^21^ proposed a distribution smoothing strategy that considers the similarity between nearby targets in both label and feature spaces to improve regression accuracy. While those methods effectively handle imbalanced datasets, they struggle with distributions with very long tails, as in precipitation data, and also implements an auxiliary layer to model the histogram. In any case, for those losses, it is numerically still more advantageous to the optimization to focus on more frequent values and ignore the less common errors from the tails.

Another general approach for handling imbalanced datasets consists of using focal losses^30,31^, which are functions that often implement a modulating term, commonly the cross-entropy, to enable learning to focus on hard negative examples. Lu et al.^32^ propose a shrinkage loss to enhance control on the focus samples and penalize the importance of easy samples while keeping the loss of hard samples unchanged. They report improvements compared to the original focal loss that penalizes both the easy and hard samples. Focal losses are very efficient in handling class imbalance, but they also struggle to tackle data imbalance within samples, as in precipitation data. In this sense, histogram-based and focal losses are not inherently adequate for our problem. We propose to address this by quantizing the reconstruction losses, which would take errors on individual quantiles equally into account when training the model.

**Figure 1:**
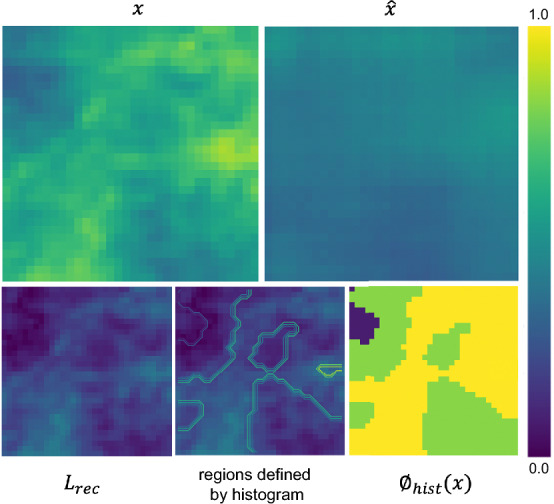
Illustration to support our quantized loss formulations. At top-left the original input image *x*, at right the reconstructed version $\hat{x}$. At bottom-left, the usual mean squared error between *x* and $\hat{x}$. In the center, we show different regions defined by the batch histogram from which the MSE is computed for both $L_{rec_{qt}}$ and $L_{rec_{wqt}}$. At bottom-right, we observe the $\phi _{hist}$ focal modulating term used to build quantized focal losses, which penalizes regions depending on how frequent the values they hold are.

## Method

The VAE defines a joint distribution between the input data space $x\in \mathcal {X}$ and the latent space $z\in \mathcal {Z}$. Commonly, we assume a simple prior distribution *p*(*z*) over the latent variables, such as a Gaussian *N*(0, *I*)^18^. The data generation process consists of two steps: (1) a value *z* is sampled from the prior *p*(*z*); (2) a value *x* is generated from a conditional distribution $p_{\theta }(x|z)$ (also usually assumed to be Gaussian), which is parameterized by a neural network: the *decoder*^17,18^. So, given a code *z*, the decoder produces a distribution over the possible corresponding values of *x*^17^.

The posterior $p_{\theta }(z|x) = p_{\theta }(x|z)p(z)/p_{\theta }(x)$, on the other hand, is often intractable, as is the optimization of the likelihood $p_{\theta }(x)$. The proposed solution in the VAE framework is to approximate the intractable posterior with $q_{\phi }(z|x)$, again assumed to be Gaussian. A neural network, the *encoder*, parameterizes $q_{\theta }(z|x)$: given a sample *x*, the encoder produces a distribution over the possible *z* values from which *x* could have been generated^17^.

The standard VAE^17,18,23^ loss to be minimized has two components, namely, a reconstruction term $L_{rec}$ and a regularization term $L_{reg}$:

1 $$\begin{aligned} \mathcal {L}(x) = L_{rec}(x) + L_{reg}(x) \equiv - \mathbb {E}_{q_{\phi }(z|x)}[\text {log}\, p_{\theta }(x|z)] + D_{KL}(q_{\phi }(z|x)||p(z)) \end{aligned}$$

where $D_{KL}$ is the KL divergence between the approximate posterior $q_{\phi }(z|x)$ and the prior *p*(*z*). The $L_{rec}$ term maximizes the log likelihood of observing data point *x* given its inferred latent variables^18^. As $p_{\theta }(x|z)$ is frequently assumed to be Gaussian, $L_{rec}$ is often the mean squared error (MSE) between samples *x* and their reconstructions $\hat{x}$^17,23^.

Subsequent works^18–20^ have investigated alternative regularization approaches to alleviate the negative impact that the KL divergence might have in the decoder reconstruction performance. InfoVAE^18^ rewrites the VAE objective and explores other divergences, such as Maximum Mean Discrepancy (MMD)^33^, that presented the best results. MMD measures the distance between two distributions by comparing their moments using kernel embeddings. We follow this idea and use the MMD as the regularization loss in our experiments.

We describe the proposed quantized reconstruction losses below as well as the focal losses we used as baselines for comparison.

### Quantized reconstruction losses

In the first approach, we propose penalizing the reconstruction loss according to the observed values’ frequency by quantizing the reconstruction loss and averaging the reconstruction losses for each quantile. Formally:

2 $$\begin{aligned} L_{rec_{qt}} = \sum _{j}^{B} \frac{1}{|\Omega _j|} \sum _{i \in \Omega _j}^{} ||x_i-\hat{x_i}|| \end{aligned}$$

where $\Omega _j$ is the set of pixel indices whose values are inside a given bin $b_j$ considering the input data histogram *h*(*x*).

The second approach slightly differs from the first one and weights the quantized reconstruction losses by the inverse likelihood of a given value based on the histogram frequency distribution. We define a histogram-based penalty function, $\omega _j$, that weights the losses depending on its frequency value. Formally:

3 $$\begin{aligned} L_{rec_{wqt}} = \sum _{j}^{B} \frac{1}{|\Omega _j|} \sum _{i \in \Omega _j}^{} \omega _j(x_i)\cdot ||x_i-\hat{x_i}|| \end{aligned}$$

where $\omega _j|j\in B$ is the histogram-based penalization function:

4 $$\begin{aligned} \omega _j(x_i) = 1 - h_j(x_i) \end{aligned}$$

Here, *h*(*x*) is the normalized histogram of a given input data *x* considering *B* bins, where the normalization consists of dividing the bin counts by the maximum bin count observed for *x*. $h_j(x) \in [0,1]$ gives the normalized frequency for a pixel value regarding a bin $b_j$.

We derive four VAE models for experimenting with these two quantized reconstruction losses by either replacing the reconstruction loss with its quantized version or adding the quantized version to the standard MSE loss. Formally, the VAE losses we derive are:

5 $$\begin{aligned} \mathcal {L}_{\{qt,wqt\}} = L_{rec_{\{qt,wqt\}}} + L_{reg} \end{aligned}$$

6 $$\begin{aligned} \mathcal {L}_{\{rec+qt,rec+wqt\}} = L_{rec} + L_{rec_{\{qt,wqt\}}} + L_{reg} \end{aligned}$$

Figure 1 shows an example of $L_{rec}$ and the regions to compute $L_{rec_{\{qt,wqt\}}}$ for a given sample. While $L_{rec}$ considers merely the pixel mean squared error between the sample and its reconstruction, $L_{rec_{\{qt,wqt\}}}$ define different regions to compute the pixel mean squared error and derive the average value comprising all regions evaluated. In this sense, errors in less frequent values are equally important for computing the final reconstruction loss.

#### Reconstruction focal losses - baselines

Although the literature presents some approaches for imbalanced regression, many are not directly applicable to the VAE framework, as they require adding an extra layer^21^ or a specific format for the output layer^29^. However, the regression focal loss presented by Yang et al.^21^ can replace the MSE in the VAE objective without additional requirements. The authors propose a regression loss inspired by the focal loss for imbalanced classification problems^30^. The scaling factor is a continuous function that maps the absolute error into [0, 1]. More specifically, the regression focal loss based on the MSE can be written as:

7 $$\begin{aligned} FL_{MSE} = \mathbf {\sigma }(\beta |x-\hat{x}|)^\gamma \cdot ||x-\hat{x}|| \end{aligned}$$

where $\mathbf {\sigma }$ is the sigmoid function, $\beta$ and $\gamma$ are hyperparameters. The intuition behind the regression focal loss is that samples with minor regression errors will contribute less to the total error than those with higher regression errors, which can help training from imbalanced datasets. Here, we propose another focal loss baseline with a modified modulating term, $\phi _{hist}$, that considers histogram imbalance. The idea is to have a baseline that also incorporates the histogram to compare with our quantized losses. Figure 1 shows an example of $\phi _{hist}$ applied to a given sample, and we observe higher quantized focus associated with less frequent values and lower quantized focus associated with more frequent values.

The quantized focal loss approach replaces the sigmoid term in Eq. (7) by $\phi _{hist}$, which depends solely on the frequency of values in a batch. Formally:

8 $$\begin{aligned} FL_{hist} = \phi _{hist}(x)^{\gamma } * ||x-\hat{x}|| \end{aligned}$$

where $\phi _{hist}(x)$ is formally defined by:

9 $$\begin{aligned} \phi _{hist}(x_i) = \omega _j(x_i) | i \in \Omega _j, j \in B \end{aligned}$$

where $\Omega _j$ is the set of pixel indices which values are inside a given bin $b_j$, and $\omega _j$ histogram-based penalization corresponding to bin $b_j$, as defined in Eq. (4).

We then experiment with two additional VAE baseline models by replacing $L_{rec}$ with each of the baseline reconstruction focal losses. Formally:

10 $$\begin{aligned} \mathcal{F}\mathcal{L}_{\{MSE, hist\}} = \lambda \cdot FL_{\{MSE, hist\}} + L_{reg} \end{aligned}$$

where $\lambda$ is a factor that controls the relative importance of the focal losses compared to the regularization loss $L_{reg}$. In our preliminary experiments, we noticed that, for the quantized losses, reconstruction and regularization terms had a similar scale, so it was not required to balance them with weights. However, for the focal losses, with $\lambda =\text {1.0}$, they showed a significantly small signal compared to the regularization term, and convergence was poor. Therefore, we set $\lambda =\text {100}$ in the focal losses experiments.

## Experiments

Our experiments examine precipitation field synthesis to evaluate how the proposed reconstruction loss quantization helps synthesize very infrequent values better. We highlight that the losses are applicable to other imbalanced datasets and encourage the reader to explore them for different applications.

### Dataset

**Figure 2:**
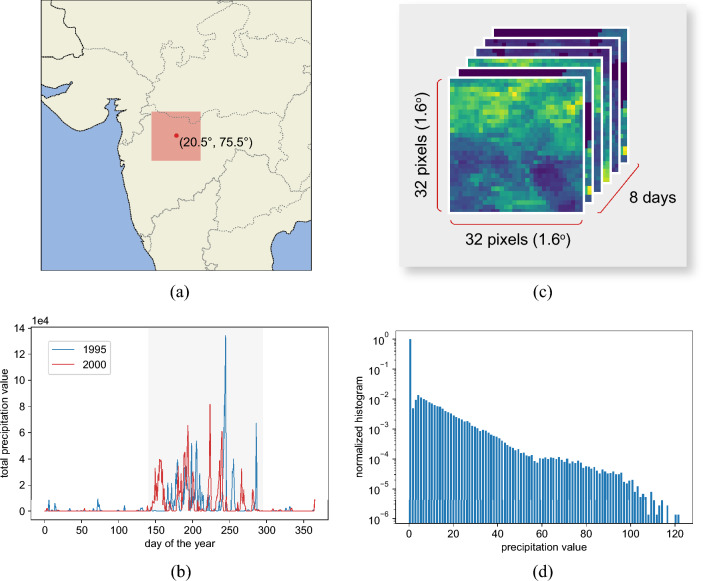
(**a**) Region of interest in the north of Maharashtra state, India. (**b**) Total daily precipitation in the region of interest for two different years: 1995 and 2000. The gray area highlights the Monsoon period, extending from around day 150 and 300. (**c**) Typical training sample from the Monsoon period, which comprises a sequence of 8 days of precipitation data in a 32$\times$32 pixels tile that corresponds to 1.6$^{\circ }\times$1.6$^{\circ }$. (**d**) The normalized histogram of precipitation values over the region of interest for year 1995; the log scale was used for better visualization. The graph shows the severe data imbalance: several days with no rain (first bin) and bins extending to very high precipitation values (>120 mm/day).

We used the Climate Hazards group Infrared Precipitation with Stations (CHIRPS) dataset^34^, a global interpolated dataset of daily precipitation providing a spatial resolution of 0.05$^{\circ }$. The data ranges from the period 1981 to the present. We experimented with a 2.4$^{\circ }\times$2.4$^{\circ }$ bounding box centered in the latitude and longitude coordinates (20.5$^{\circ }$, 75.5$^{\circ }$), in Maharashtra state, India, as indicated in Fig. 2a. We used daily precipitation data from 1981 to 2010 for the training set and from 2011 to 2019 for the test set.

Figure 2b shows the total daily precipitation in the region of interest for two randomly selected years. As indicated by the gray area in Fig. 2b, the monsoon period begins around day 150 and continues until about day 300. We considered sequences of 8 days only in this time range for each year and bounding boxes of 1.6$^{\circ }\times$1.6$^{\circ }$ to generate training and test samples. More specifically, we use a sliding window mechanism to crop bounding boxes of 32$\times$32 pixels inside the 48$\times$48 pixels area highlighted in red in Fig. 2a. Therefore, the samples have 32 pixels $\times$ 32 pixels $\times$ 8 days, as seen in Fig. 2c. The final training set contains 26,100 examples and the test set, 9000. The datasets are rescaled to the range [0, 1].

As observed in Fig. 2d, the precipitation data is severely imbalanced: while dry days are frequent over the year, heavy rain or extreme precipitation events are rare. This intrinsic imbalance characteristic is especially challenging for machine learning models: it is difficult to learn the extreme events effectively when only a few examples are available. On the other hand, efficient risk analysis for extreme weather events depends on reliable data prediction and synthesis.

### Experimental design

**Figure 3:**
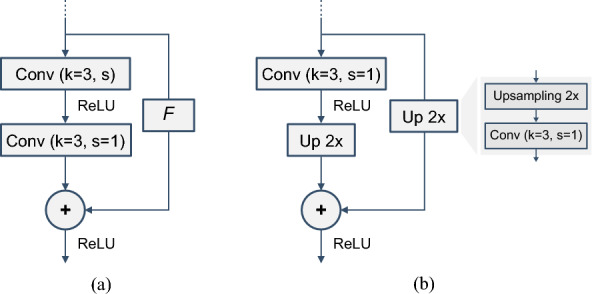
(**a**) Residual unit, similar to ResNet18 units; kernels (*k*) have size 3. *F* can be the identity function when feature map shapes for addition match or a 1$\times$1$\times$1 convolution when the shapes are different. The first convolutional layer can have stride (*s*) 1 or 2, while the second always has *s* = 1. (**b**) Residual unit with upsampling. The *Up 2x* function comprises a 2$\times$ upsampling (nearest neighbor interpolation) followed by a convolution with *k* = 3 and *s* = 1.

Table 1 presents the encoder and decoder networks we used for the 3D VAE. The encoder architecture is based on a ResNet18^35^: we replaced the 2D convolutions with the 3D version but kept the same type of residual units (see Fig. 3a). We removed the last two residual units compared to the original ResNet18 because our input size is much smaller than the 112$\times$112 pixels images used in the original case. After the last residual unit, we have two dense layers for optimizing $\mu _x$ and $\sigma _x$, which are used to sample *z* following a standard normal distribution. The decoder receives an input array *z* with the size of the latent space dimension that is ingested to a dense layer to be reshaped into 256 activation maps of size $8\times 8\times 2$. These maps serve as input to consecutive residual units that can be equal to the ones used in the encoder (Fig. 3a) or residual units with upsampling (Fig. 3b), that increase the data size by two in the spatial and temporal dimensions. The decoder has a total of two residual upsampling units to increase the $8\times 8\times 2$ maps back to the original size of $32\times 32\times 8$. A convolution using one filter with sigmoid activation delivers the final output.

We used the Adam optimizer with beta1 as 0.9, and beta2 as 0.999. We used a learning rate of 5$\cdot \text {10}^{-\text {5}}$ for the quantized losses and 1$\cdot \text {10}^{-\text {5}}$ for the others. In the loss functions based on histograms, the number of bins was experimentally set to 100. We used $\gamma =\text {1}$ and $\beta =\text {0.2}$ as in the original paper^21^.

We trained the models for 100 epochs, with 32 data samples per batch, and monitored the total validation loss to apply early stopping. We set initial seeds for Tensorflow and NumPy libraries to allow a fairer comparison between different trained models and enable our tests’ reproducibility. All experiments were carried out using V100 GPUs.

**Table 1:** Encoder and decoder architectures.

Encoder		Decoder	
Layer	Output shape	Layer	Output shape
Conv s=1	$32\times 32\times 8\times 64$	Dense	32, 768
ReLU	$32\times 32\times 8\times 64$	Reshape	$8\times 8\times 2\times 256$
Res s=1	$32\times 32\times 8\times 64$	Res s=1	$8\times 8\times 2\times 256$
Res s=1	$32\times 32\times 8\times 64$	Res s=1	$8\times 8\times 2\times 256$
Res s=2	$16\times 16\times 4\times 128$	Res s=1	$8\times 8\times 2\times 128$
Res s=1	$16\times 16\times 4\times 128$	Res up	$16\times 16\times 4\times 128$
Res s=2	$8\times 8\times 2\times 256$	Res s=1	$16\times 16\times 4\times 64$
Res s=1	$8\times 8\times 2\times 256$	Res up	$32\times 32\times 8\times 64$
Flatten	32, 768	Conv s=1	$32\times 32\times 8\times 1$
Dense $\mu _x$	30	Sigmoid	$32\times 32\times 8\times 1$
Dense $\sigma _x$	30		

### Metrics

The VAE learns to generate stochastic samples of precipitation fields from the latent variable ${\textbf {z}}\sim N(0, I)$. To use the VAE after training, we can sample from the latent normal distribution and then use the decoder to generate precipitation fields. There are two critical aspects to evaluate in a VAE: the quality of the samples (associated with the reconstruction loss) and the variability of the synthetic data (associated with the regularization loss). In this work, as we propose losses to improve sample quality, especially for rare values, we focus on evaluating synthesis quality. A straightforward way to verify that the latent space encodes relevant information that can be decoded into meaningful samples is to analyze the *reconstruction quality*. We take input samples from the real data, use the encoder to map them to the latent space, and compare the decoded outputs with the input data. If properly trained, the reconstructed samples should be similar to the input data. Notice, however, that one should not expect perfect reconstructions, as the regularization term usually indirectly penalizes reconstruction quality for allowing regular stochastic sampling and data synthesis.

For reconstruction evaluation, we need metrics that can represent not only the difference between input and reconstructed samples but also that can inspect how this difference spreads across precipitation values of lower and higher frequencies. We then selected two metrics: quantized mean-squared error and QQ (quantile-quantile) plots.

The average MSE provides information about the overall reconstruction quality, with lower MSE values associated with more similar images or better reconstruction. The quantized MSE is related closely to $L_{rec_{qt}}$: we compute the average MSE for each histogram bin, i.e. considering only pixels that fall into each particular bin. We derive graphs showing the average MSE values for each bin, so one can visually inspect how the reconstruction quality relates to how frequent the values are in the known data histogram.

A QQ plot is a graphical method for comparing probability distributions with a reference distribution. In QQ plots, distribution quantiles are plotted against each other, which means that a point in the graph corresponds to one quantile from a given distribution plotted against the same quantile in another distribution. In our case, the pixel values of the input samples represent the reference distribution (x-axis), and the pixel values of their respective reconstructions define the other distribution (y-axis). If these two distributions are similar, the points will fall approximately on the line where the x-axis equals the y-axis (45$^{\circ }$ straight line). If the distributions are linearly related, the quantiles will fall approximately on a straight line but not necessarily on the 45$^{\circ }$ line.

## Results and discussion

In total, we evaluated seven VAE models, each of them with a different loss configuration. In our results, we name the models according to their losses, as presented in the Method section, meaning that L_rec corresponds to the model trained using $\mathcal {L}$, L_qt using $\mathcal {L}_{qt}$, FL_hist using $\mathcal{F}\mathcal{L}_{hist}$ and so on.

**Figure 4:**
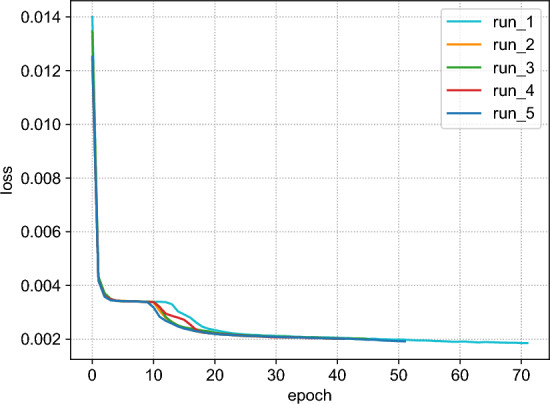
Training loss for five runs of the model trained with the baseline loss $\mathcal {L} = MSE + MMD$. Each run corresponds to a different random seed, keeping the training configuration unchanged.

Although we adopted an encoder-decoder based on a standard architecture (ResNet18), in order to verify its stability, we conducted the following experiment: we trained the model using the baseline loss L_rec five times, using different random seeds and the configuration detailed in the previous section. Figure 4 depicts the training losses of these five runs and shows that the model converges to very similar values in all of them.

**Figure 5:**
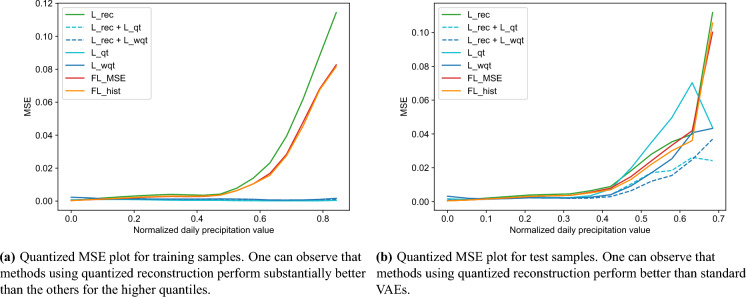
Quantized MSE plot for training and tet samples.

**Figure 6:**
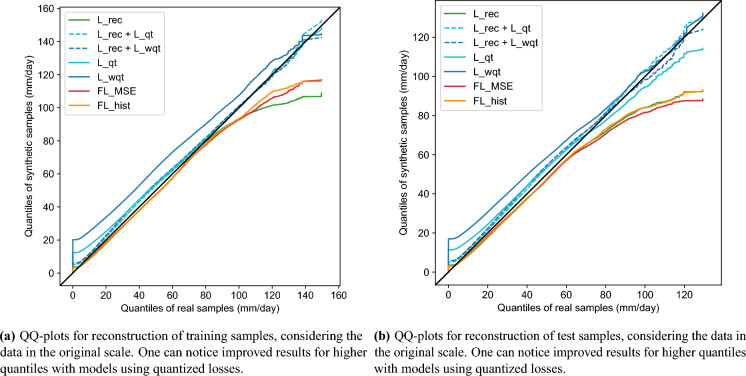
QQ-plots for reconstruction of training and test samples, considering the data in the original scale.

**Figure 7:**
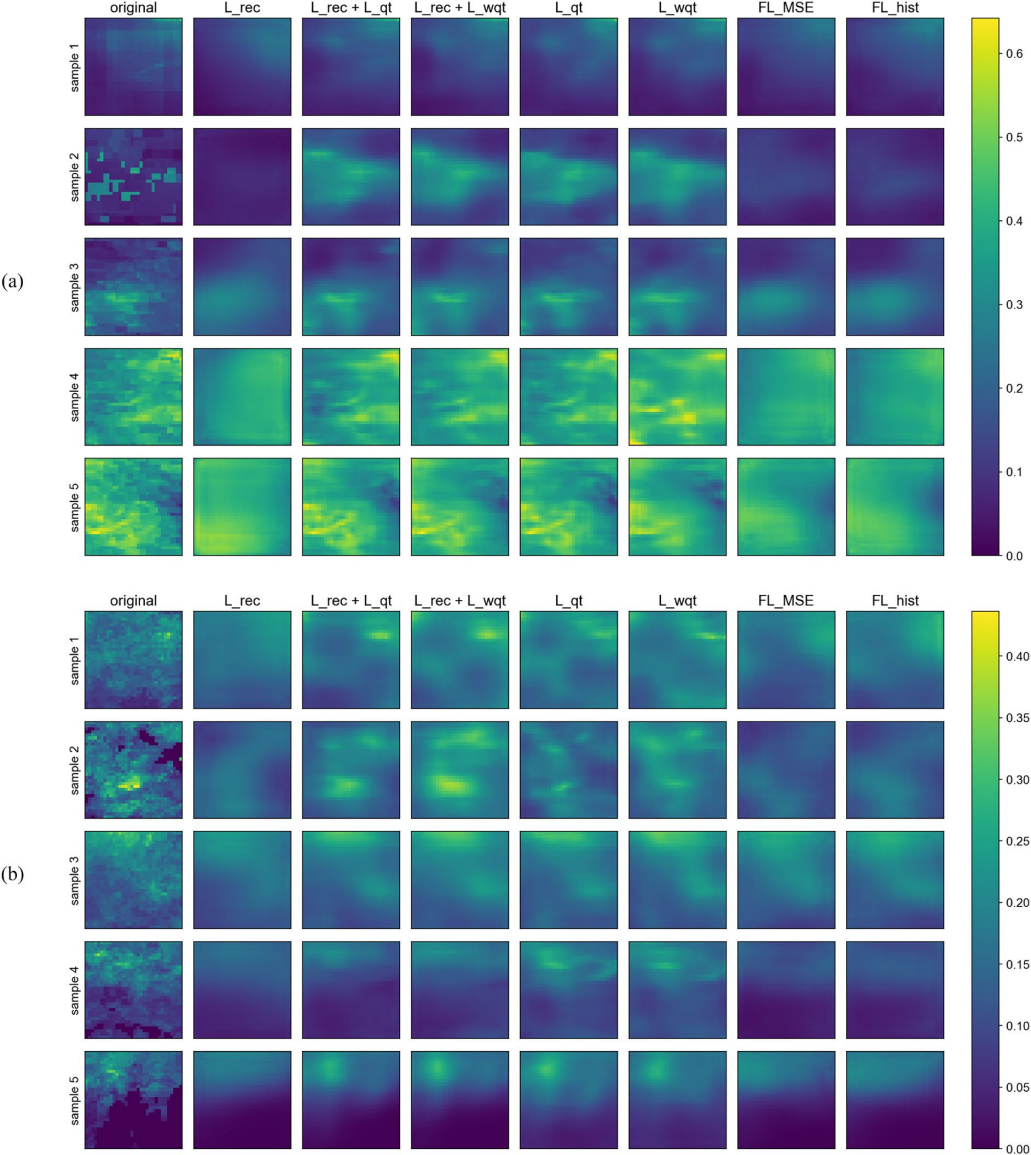
Examples of reconstructions of samples from the training set (**a**) and test set (**b**), where precipitation is lower in blueish pixels and higher in yellowish ones. Rows represent different weather fields selected at random between the samples with higher maximum precipitation, and columns represent the various models tested.

We can observe from Fig. 5a that reconstruction of very infrequent values in the training set is challenging for most trained models. However, models using $L_{rec_{qt}}$ or $L_{rec_{wqt}}$ (blue lines) performed much better than the other models for the higher quantiles, which indicates a significant benefit in quantizing the losses considering the values’ frequency. On the other hand, the focal loss models performed marginally better than the standard VAE (L_rec) for the higher quantiles. Considering the extreme values (right side of the graph in Fig. 5a), the difference between the MSE of the quantized losses and the reference can reach two orders of magnitude (${10}^{-3}$ for quantized losses and ${10}^{-1}$ for L_rec).

Regarding the reconstruction of unseen test samples, the performance is, as expected, worse than that observed for the training set (see Fig. 5b). Still, the models with quantized losses show a significant quality improvement for infrequent values compared to the others, in this case, with a difference of around one order of magnitude in MSE compared to L_rec in the highest bins. Figure 5 also shows that models L_qt and L_wqt (solid blue lines) presented higher MSE values for the lower and higher quantiles than the other quantized models (dashed blue lines). This result indicates that combining the quantized losses with the original MSE loss can improve the quality of rare values while keeping good performance for the frequent values.

The QQ-plot between input and generated samples provides information about how well the model respects the original distribution. Figure 6a shows the QQ-plot for the training data reconstruction, where the x-axis represents the quantiles of the input samples. The model with standard MSE loss (green line) presents quantiles similar to the reference distribution until the value of 80mm/day, from which the model underestimates the quantiles considerably. This behavior shows that, with this loss, the model cannot represent the entire training distribution, failing to generate the more extreme samples. The focal loss models demonstrate a similar behavior but with marginally better performance for higher quantiles. The models with quantized losses overestimate the quantiles in the lower region (more frequent values) and show quantile matching for the higher values. Specifically, model L_wqt overestimates the quantiles over almost the entire range, while L_qt has higher discrepancies only in the region around zero. Again, the models that combine the quantized losses and regular MSE (dashed blue lines) present the best results for the higher quantiles with only a small overestimation for lower values, indicating that this combination can lead to more robust models. We highlight that the models that fail to generate higher precipitation are not adequate in the weather generator context, as the extreme events are usually the ones of more interest.

We observe similar behaviors for the unseen test data reconstruction (Fig. 6b): the quantization significantly improved the performance over the standard VAE in the higher and less frequent precipitation values. The focal losses, however, present equal (FL_hist) or lower (FL_MSE) performance than the standard VAE.

Finally, we present a visual comparison of randomly selected input samples and their respective reconstruction for each model to help illustrate the discussion above. In Fig. 7a, one can see five different days selected from the training dataset in each row and their reconstructions in the columns. The models using quantized losses present the most appealing visual results, with reasonable outputs even in the case of a low-quality input sample (second row). It is also important to notice that the spatial characteristics are reasonably represented: the location of higher precipitation events is preserved. The standard VAE and focal loss models show smoother reconstructions and fail to reproduce higher values, confirming the previous observations on the quantized MSE charts.

Figure 7b shows a similar comparison for test samples, also randomly selected. As expected, the results are worse for all models (smoother reconstructions) than for the training set case. However, the quantized models still perform significantly better than the focal losses or standard VAE, preserving some higher precipitation values.

We report improvements when using quantized losses over standard reconstruction losses for VAE, handling the reconstruction quality from an imbalanced data perspective. Considering focal losses were designed to improve models in imbalanced datasets, one would expect competitive performance in our experiments, and conversely, we observed that they are, in best cases, only marginally better than L_rec. We argue that, in reality, focal losses are not very fit for datasets composed of samples with highly imbalanced histograms, such as ours. More specifically, FL_MSE further penalizes the error by powering the error value itself, assuming that samples with rare values will have high associated errors. However, in our dataset, even days with rare, heavy precipitation events contain several points with values close to zero (see Fig. 7). Considering that our sample is a 3D volume of pixels and that the associated error is an average computed over all the pixels, pixels with low precipitation values smoothen out the smaller amount of pixels with high error values associated with rare events. In that case, the difference between the average MSE of common and rare samples will be relatively small, and the focal loss penalization will not distinguish those errors properly. Concerning FL_hist, it uses the histogram to penalize the MSE, but the same effect happens, as the error is computed over the entire 3D sample. On the other hand, the quantized losses calculate the MSE for each bin (with penalization) in a given sample and then average those errors, which prevents smoothening rare event pixel errors for each individual sample.

### Stochastic synthesis

We further present a qualitative analysis for stochastic data synthesis, considering the model L_rec + L_wqt, which delivered good results for the quantized MSE and QQ-plots.

**Figure 8:**
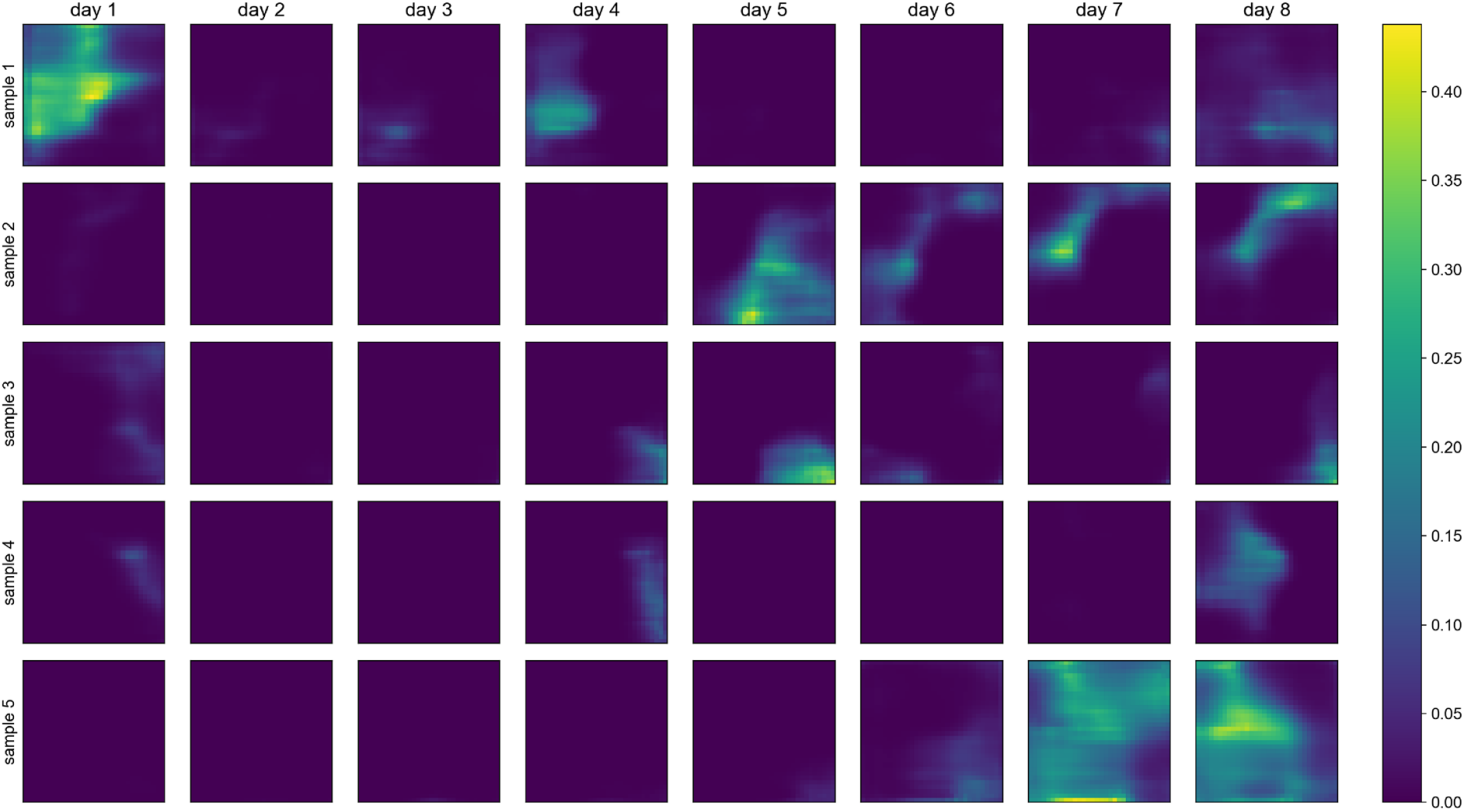
Random samples. Columns represent each day in the 8-day samples and each row represent a different sample.

As mentioned in the Metrics section, one can generate stochastic samples from a trained VAE by random sampling from the normal distribution in the latent space and ingesting such data into the decoder to create the corresponding synthetic data. In Fig. 8, we show five random precipitation samples from the model L_rec + L_wqt, obtained as described above, and observe they present some noticeable variability, which points to successful training. Additionally, some samples show high precipitation values (at the order of 0.4, which is relevant considering the precipitation range value and the normalization between 0 and 1.0). Random samples generated using the trained L_rec model do not present such high precipitation values.

**Figure 9:**
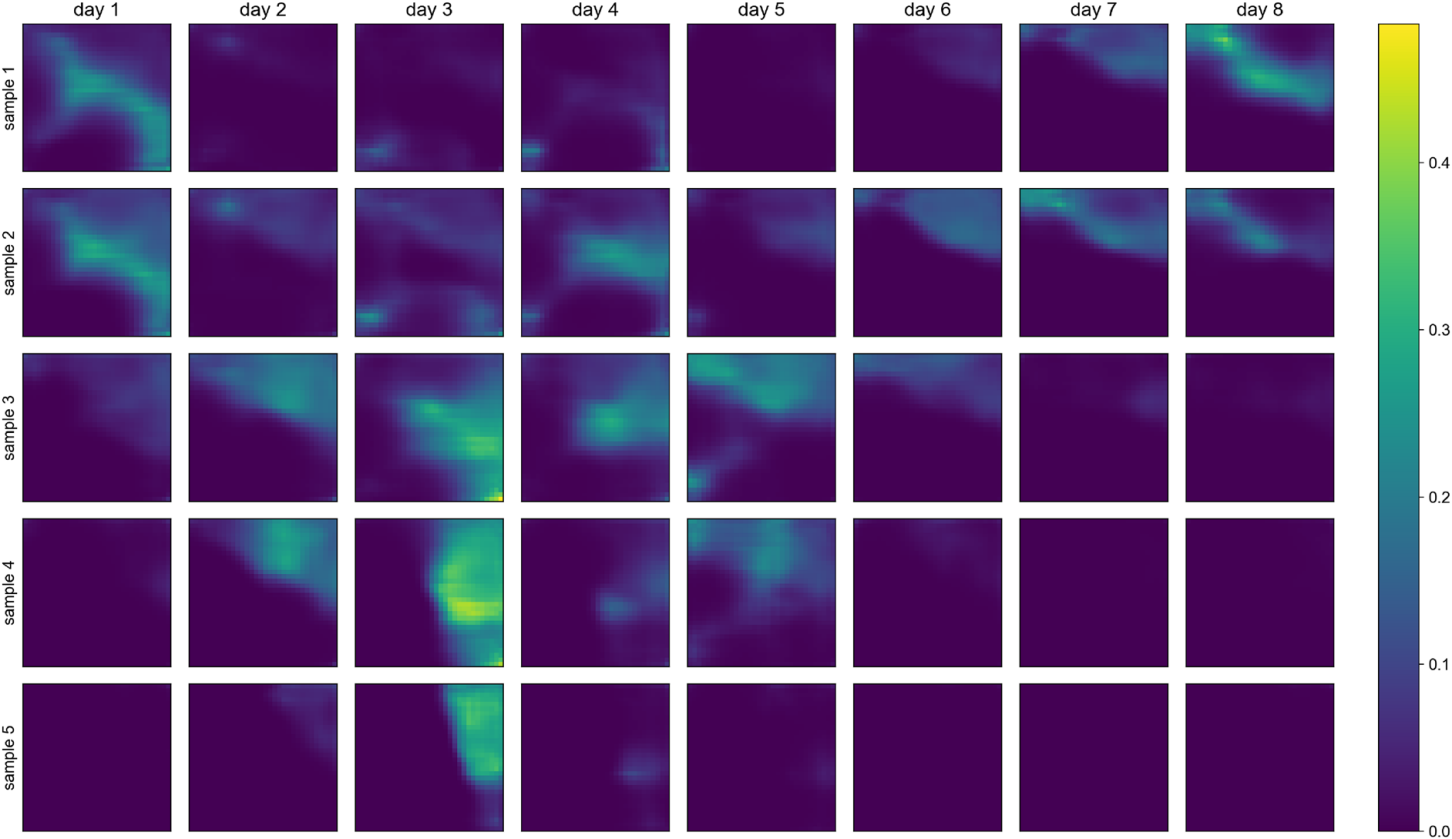
Latent space interpolation. We selected 2 random latent space points (sample 1 and sample 5) and other 3 samples in between them in a regularly spaced grid. Columns represent each day in the 8-day samples and each row represent a different sample.

We also experimented with latent space interpolation, a qualitative test to verify if the latent space has proper regularization. We first select two random samples from the latent space normal distribution. Then, we create a regular grid between them, generating three samples in between the first two points, with each dimension regularly spaced. For example, if for the first dimension of ${\textbf {z}}$, the two random samples were 0.1 and 0.5, respectively, then the other three samples for this dimension would be 0.2, 0.3, and 0.4. Each latent code in the regular grid is then decoded with the trained decoder. Figure 9 shows this experiment for the model L_rec + L_wqt, and we can see that sample 1 (top row) gradually transforms into sample 5 (bottom row), which is the expected behavior for a well-regularized latent space.

**Figure 10:**
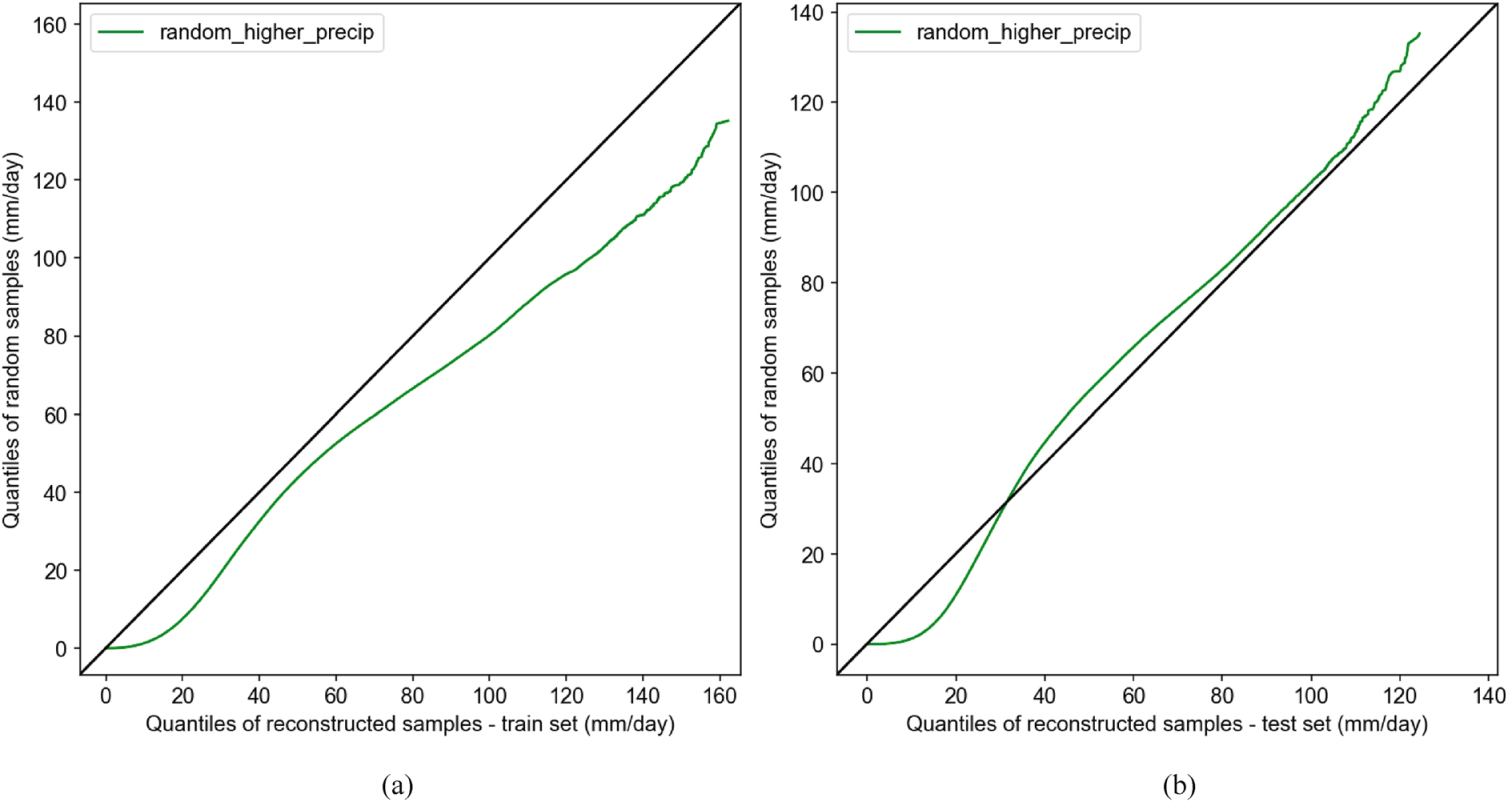
QQ-plots of random samples against reconstructed samples from the (**a**) train set and from the (**b**) test set. We only considered samples that have at least 1 pixel with precipitation value above the quantile 0.99 of the training set.

In Fig. 10, we compare the values distribution of random and reconstructed samples with higher precipitation values. The idea is to compare whether the distribution of values in samples with higher precipitation values for random samples is comparable to the reconstructed ones. For this analysis, we considered only samples with at least one pixel higher than the quantile 0.99 of the training set (0.2219). To generate the QQ-plots, we then selected the 2 000 samples from each set with the highest sums of precipitation values. We use this selection scheme to compare only samples with extreme values (localized in one pixel or spread across the region). Next, we use the trained L_rec + L_wqt model to reconstruct the selected samples from the train and test sets (x-axis in Fig. 10a and b, respectively). We use the decoder to generate 10,000 random precipitation samples and the scheme above to select 2000 for comparison. One can observe that Fig. 10 presents a very consistent distribution of random and reconstructed precipitation values, especially when the reconstruction involved the test set. The fact that the QQ-plots are more consistent for the test set than for the training set is probably due to the first being composed of samples with slightly lower precipitation values than the latter (in our dataset the last 10 years were less rainy). Most importantly, higher quantiles seem to be well represented in the stochastically generated samples regarding the real samples’ distributions observed in both training and testing sets.

## Conclusions

This paper introduced new quantized reconstruction losses to help variational auto-encoders better synthesize extreme weather fields. We proposed embedding histogram-based penalization into commonly used reconstruction loss to modify the loss depending on how probable a value is. We also explored focal losses using a similar conceptualization to modify the focus-modulating term and privilege loss in less frequent values.

Our results show that including quantization in regular and focal losses improved trained models’ performance for reconstructing extreme weather fields consistently compared to those not using histogram-based penalization.

Evaluating the mean-squared error between the input and its corresponding reconstruction pointed to a solid improvement of models using quantized losses. Compared to regular VAEs for regression, models using a combination of quantized and standard reconstruction losses presented overwhelming results by penalizing errors associated with less frequent values and, at the same time, sticking to the standard MSE to reduce pointwise errors.

Future work involves testing different regularization terms combined with quantized reconstruction losses to evaluate VAE-based stochastic data synthesis with very imbalanced histograms. Considering the specific use case of weather generators, we envisage exploring different architectures for encoders and decoders to improve the reconstruction towards sharper outcomes.

## Data Availability

The datasets generated and/or analysed during the current study are available in the Climate Hazards group Infrared Precipitation with Stations (CHIRPS)^34^ repository, https://www.chc.ucsb.edu/data/chirps.
